# Dietary and Nutritional Considerations for Metabolic Dysfunction-Associated Steatotic Liver Disease in Psoriasis and Hidradenitis Suppurativa: A Narrative Review

**DOI:** 10.3390/nu18193253

**Published:** 2026-10-02

**Authors:** Woo Chiao Tay, Choon Fong Liew, Yu Jun Wong, Grace Lai-Hung Wong, Hazel H. Oon

**Affiliations:** 1 National Skin Centre, 1 Mandalay Road, Singapore 308205, Singapore; hazeloon@nhghealth.com.sg; 2 Specialist Care Group, Diabetes and Endocrinology Centre, Mount Elizabeth Medical Centre, Singapore 228510, Singapore; 3 Department of Gastroenterology & Hepatology, Changi General Hospital, Singapore 529889, Singapore; 4 Duke-NUS Medical School, 8 College Road, Singapore 169857, Singapore; 5 Department of Medicine and Therapeutics, Medical Data Analytics Centre (MDAC), 9/F Prince of Wales Hospital, 30-32 Ngan Shing Street, Shatin, Hong Kong SAR, China; 6 Institute of Digestive Disease, Hong Kong SAR, China; 7 Li Ka Shing Institute of Health Sciences, The Chinese University of Hong Kong, Hong Kong SAR, China; 8 Skin Research Institute of Singapore, Singapore 308205, Singapore; 9 Lee Kong Chian School of Medicine, Nanyang Technological University, Singapore 308232, Singapore; 10 Yong Loo Lin School of Medicine, National University of Singapore, Singapore 117597, Singapore

**Keywords:** metabolic dysfunction-associated steatotic liver disease, psoriasis, hidradenitis suppurativa, Mediterranean diet, nutrition, weight loss, incretin therapy, dermatology

## Abstract

Metabolic dysfunction-associated steatotic liver disease (MASLD) is increasingly recognised in patients with chronic inflammatory skin disease, particularly psoriasis, for which epidemiological evidence of increased MASLD prevalence is relatively strong; corresponding data in hidradenitis suppurativa (HS) remain more limited. This narrative review examines the epidemiological and biological links between psoriasis, HS and MASLD, and evaluates dietary and nutritional strategies relevant to their multidisciplinary management. Particular emphasis is placed on distinguishing direct evidence in patients with concomitant inflammatory skin disease and MASLD from evidence derived from dermatology cohorts without liver-specific outcomes and from general MASLD populations. Mediterranean-style dietary patterns, energy restriction and weight loss, improved diet quality, and reduced intake of ultra-processed foods and added sugars are supported principally by MASLD and cardiometabolic data, with more limited direct evidence in psoriasis or HS. The review also discusses nutritional considerations during incretin-based therapy, including reduced food intake, preservation of lean mass, micronutrient adequacy and gastrointestinal adverse effects, while highlighting areas in which recommendations remain extrapolated or expert-derived. For dermatologists, the practical emphasis is on recognition of metabolic risk, general dietary counselling and appropriate multidisciplinary referral. Overall, nutritional management is an important component of MASLD care, but dedicated intervention studies in patients with concomitant psoriasis or HS remain a major evidence gap.

## 1. Introduction

Metabolic dysfunction-associated steatotic liver disease (MASLD) describes hepatic steatosis occurring in the context of cardiometabolic dysfunction and encompasses a spectrum from steatosis to metabolic dysfunction-associated steatohepatitis (MASH), progressive fibrosis, cirrhosis and hepatocellular carcinoma [1,2]. Psoriasis and hidradenitis suppurativa (HS) are chronic inflammatory skin diseases that are frequently associated with obesity, insulin resistance and metabolic syndrome, providing a rationale for examining liver disease as part of their broader comorbidity profile [3,4].

The epidemiological association is best established for psoriasis. Cohort and meta-analytic data consistently demonstrate a higher prevalence of MASLD among patients with psoriasis, including prevalence estimates of approximately 43–44% in selected psoriasis cohorts compared with 22–25% in control populations [5,6,7]. By contrast, MASLD epidemiology in HS remains less well characterised, although HS shares many of the metabolic and inflammatory abnormalities implicated in steatotic liver disease [4,8]. Evidence derived from psoriasis, HS and general MASLD populations should therefore not be considered interchangeable.

Dietary modification and weight management are central components of MASLD care, yet evidence specifically addressing patients with concomitant inflammatory skin disease and MASLD remains limited. This narrative review is intended primarily for dermatologists and other clinicians involved in multidisciplinary care. It aims to summarise the epidemiological and biological links between psoriasis, HS and MASLD; evaluate dietary strategies while distinguishing direct evidence from extrapolation from general MASLD populations; review nutritional considerations associated with incretin-based therapy; and outline practical roles for dermatologists within multidisciplinary metabolic care.

### Literature Search and Evidence Framework

This narrative review was informed by targeted searches of PubMed from database inception to 1 July 2026. Searches were structured around three evidence domains: (1) the relationship between MASLD and psoriasis or HS; (2) dietary or nutritional interventions in psoriasis or HS; and (3) dietary, nutritional and incretin-based management of MASLD. The principal Boolean search concepts were: (“metabolic dysfunction-associated steatotic liver disease” OR MASLD OR “metabolic dysfunction-associated steatohepatitis” OR MASH OR “non-alcoholic fatty liver disease” OR NAFLD OR “non-alcoholic steatohepatitis” OR NASH) AND (psoriasis OR “hidradenitis suppurativa”); (psoriasis OR “hidradenitis suppurativa”) AND (diet OR nutrition OR “Mediterranean diet” OR “caloric restriction” OR fasting OR “weight loss” OR “ultra-processed food” OR micronutrient OR supplement); and (MASLD OR MASH OR NAFLD OR NASH) AND (diet OR nutrition OR “Mediterranean diet” OR “caloric restriction” OR “weight loss” OR “ultra-processed food” OR “GLP-1” OR “glucagon-like peptide-1” OR incretin OR micronutrient OR supplement).

English-language original studies, systematic reviews, meta-analyses, clinical practice guidelines and consensus statements relevant to the review objectives were considered. Where multiple publications addressed the same question, preference was given to authoritative guidelines, systematic reviews and meta-analyses, larger or more recent cohort studies, and primary randomised trials reporting clinically relevant outcomes. Reference lists of included publications were also screened. Preclinical or mechanistic studies were included selectively where they informed emerging biological pathways or experimental interventions. Given the narrative design, study selection was not conducted as a formal systematic-review process and no formal risk-of-bias assessment or quantitative evidence synthesis was performed.

To improve transparency in interpretation, evidence is considered in four broad categories throughout this review: direct evidence from patients with concomitant psoriasis or HS and MASLD; dermatology-specific evidence from psoriasis or HS populations without liver-specific outcomes; extrapolated evidence from general MASLD or metabolic populations; and mechanistic or preclinical evidence. Supplementary Table S1 summarises key dietary evidence according to source population and evidence type.

## 2. MASLD in Psoriasis and Hidradenitis Suppurativa: Epidemiology and Shared Biology

**Figure 1:**
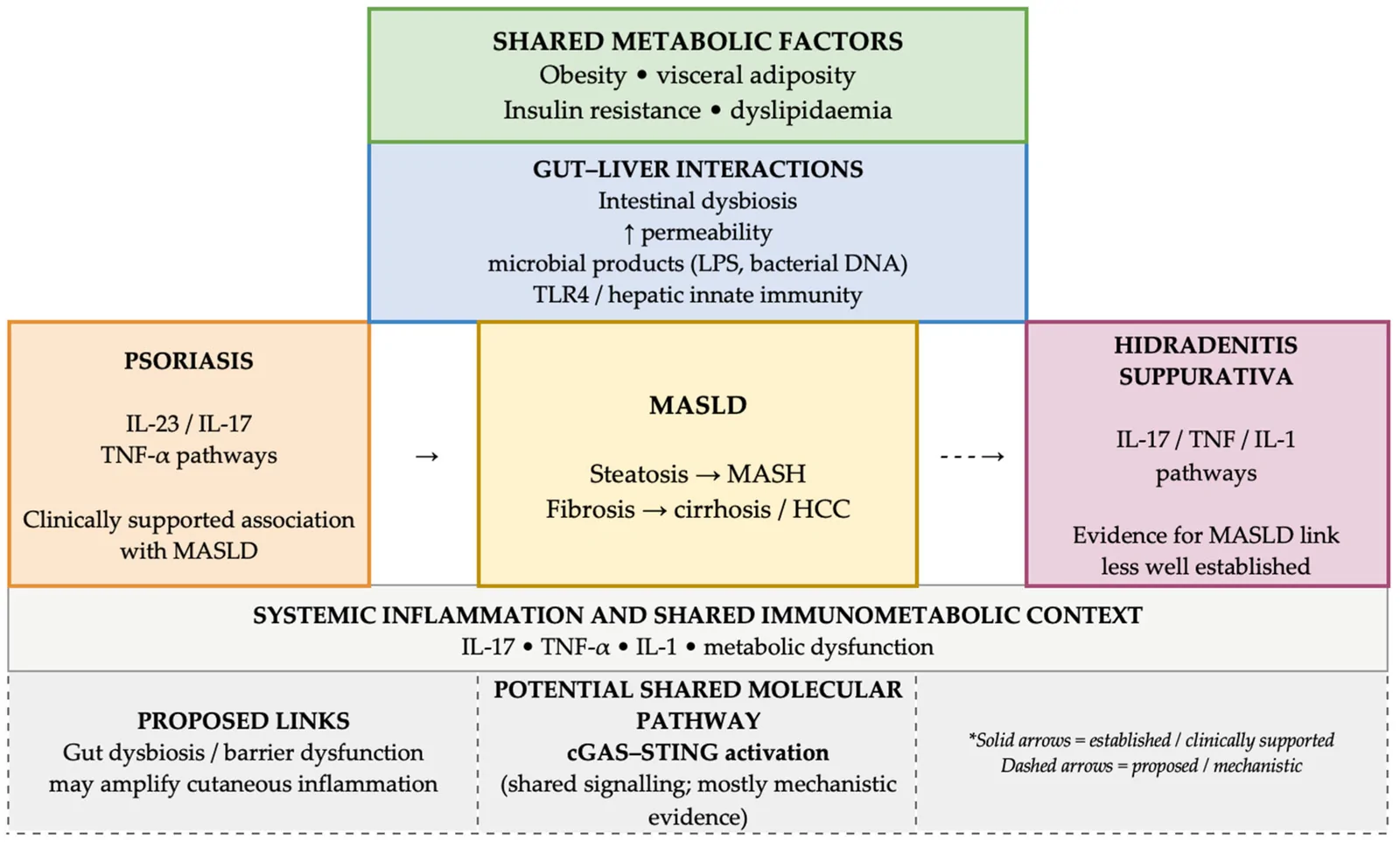
Established and proposed pathways linking psoriasis, hidradenitis suppurativa (HS), and metabolic dysfunction-associated steatotic liver disease (MASLD). Solid arrows (–––) indicate established or clinically supported relationships, including shared metabolic risk factors and the gut–liver axis in MASLD. Dashed arrows (- - - -) indicate proposed or predominantly mechanistic relationships, including microbiome-mediated effects on cutaneous inflammation and shared cGAS–STING signalling. The association between psoriasis and MASLD is better supported than that between HS and MASLD.

The established and proposed pathways linking psoriasis, HS, and MASLD are summarised in Figure 1, with solid arrows indicating clinically supported relationships and dashed arrows indicating predominantly mechanistic or proposed associations.

### 2.1. Epidemiology and Metabolic Risk

The association between psoriasis and MASLD is supported by an increasing body of epidemiological evidence. Meta-analytic and cohort studies demonstrate a higher prevalence of MASLD among patients with psoriasis than among individuals without psoriasis [5,6,7]. Patients with concomitant psoriasis and MASLD also exhibit a greater burden of metabolic abnormalities, including higher body mass index, waist circumference, triglyceride levels and C-peptide concentrations, together with lower high-density lipoprotein cholesterol [6]. Psoriasis severity alone does not appear to account fully for hepatic involvement; metabolic dysfunction and systemic inflammatory burden are likely to contribute substantially.

MASLD may also occur in patients with psoriasis who do not have obesity. Lean MASLD has been reported more frequently in psoriasis cohorts than in controls [9,10]. This is particularly relevant in Asian populations, in whom metabolic risk may occur at lower body mass index values. A BMI of 23 kg/m^2^ is commonly used as a threshold for increased metabolic risk in Asian populations and should not be interpreted as a diagnostic threshold for MASLD [10,11]. Normal aminotransferase levels also do not exclude steatotic liver disease or clinically relevant fibrosis [7].

The epidemiological evidence is considerably less developed for HS. HS is strongly associated with obesity, insulin resistance and metabolic syndrome and shares inflammatory pathways implicated in MASLD [4,8,12]. However, dedicated studies defining the prevalence, severity and natural history of MASLD specifically in HS remain limited. The association between HS and MASLD should therefore be regarded as clinically plausible but less firmly established than that between psoriasis and MASLD.

### 2.2. Shared Inflammatory, Metabolic and Genetic Pathways

Psoriasis and MASLD share several inflammatory abnormalities. Both conditions involve activation of pathways centred on interleukin (IL)-17, tumour necrosis factor-alpha (TNF-α), IL-1 and related innate and adaptive immune responses [8,13]. Comparative transcriptomic and single-cell analyses have identified overlapping inflammatory signatures involving macrophages, T lymphocytes, natural killer cells and dendritic-cell-related pathways, together with increased activity of IL-17, IL-1 and cGAS–STING-associated signalling [13]. Other immune-cell populations, including eosinophils, may be present in psoriatic lesions, although their relevance to the psoriasis–MASLD interface is uncertain [14]. These observations support overlapping inflammatory biology but do not establish a direct causal pathway between cutaneous and hepatic disease.

Metabolic dysfunction provides an additional link. Obesity, visceral adiposity, insulin resistance and dyslipidaemia are common in psoriasis and HS and are established drivers of hepatic steatosis and progression to MASH. Adipose tissue can also contribute to chronic low-grade inflammation through altered adipokine and cytokine signalling. These shared metabolic abnormalities may help explain why hepatic involvement can occur independently of the severity of cutaneous disease [8,15].

Genetic susceptibility may further modify MASLD risk, particularly in lean individuals. Variants in PNPLA3, TM6SF2 and MBOAT7 affect hepatic lipid handling and have been associated with susceptibility to steatosis and progressive liver disease [11,16]. PNPLA3 I148M impairs lipid-droplet triglyceride mobilisation, TM6SF2 influences hepatic lipid export and cholesterol metabolism, and MBOAT7 is involved in phospholipid remodelling [16]. These variants may partly explain why MASLD can develop in the absence of conventional obesity, although their specific contribution to MASLD in psoriasis or HS has not been adequately defined.

### 2.3. Gut–Liver Interactions and Proposed Links with Cutaneous Inflammation

The gut–liver axis is well established in metabolic liver disease. Dietary and metabolic stress—including lipid overload, insulin resistance, oxidative stress and mitochondrial dysfunction—can alter intestinal microbial composition and impair epithelial-barrier integrity. Increased intestinal permeability permits microbial products such as lipopolysaccharide and bacterial DNA to enter the portal circulation, where they can activate hepatic innate immune pathways including toll-like receptor 4 signalling [17]. These processes have been implicated in hepatic inflammation, insulin resistance and progression from steatosis to steatohepatitis and fibrosis, although increased intestinal permeability is not universal across all stages of MASLD.

Human observational data suggest that related abnormalities may occur in psoriasis. In a prospective study of 215 patients with moderate-to-severe psoriasis, bacterial translocation was more frequent among patients with concomitant fatty liver disease than among those without liver disease (29.7% versus 13.7%) and was associated with higher circulating inflammatory mediators [18]. Psoriasis has also been associated with intestinal dysbiosis and altered short-chain-fatty-acid-producing bacterial populations [19,20]. Gut microbial alterations have similarly been described in HS, although findings are heterogeneous and direct evidence linking these changes to MASLD in HS is lacking [19].

Innate immune pathways may provide an additional point of biological convergence. The cGAS–STING pathway is activated by aberrantly localised microbial or host DNA and can induce type I interferon and NF-κB-dependent inflammatory signalling. Experimental and bioinformatic studies implicate cGAS–STING activation in metabolic liver injury and have also identified increased pathway activity in psoriasis [13,21]. However, these data are predominantly mechanistic or associative, and equivalent evidence in HS is limited. cGAS–STING should therefore be regarded as a potential shared pathway rather than an established causal link between skin and liver disease.

Overall, current evidence supports established gut–liver interactions in MASLD and demonstrates overlapping microbial and inflammatory abnormalities in psoriasis and, to a lesser extent, HS. A distinct bidirectional “gut–skin–liver axis”, however, has not been established clinically. These mechanisms provide biological plausibility for dietary strategies that improve metabolic health and diet quality, but should not be interpreted as evidence that microbiome-directed interventions improve both hepatic and cutaneous outcomes.

## 3. Dietary Strategies: Evidence and Extrapolation

Diet and weight management are foundational components of MASLD care, but most intervention evidence has been generated in general MASLD populations rather than in patients with concomitant psoriasis or HS. Dermatology-specific studies largely address skin outcomes without liver-specific endpoints. The following sections therefore distinguish established MASLD evidence from indirect dermatological evidence.

### 3.1. Mediterranean Dietary Pattern

Mediterranean-style dietary patterns are among the dietary approaches supported by the most consistent evidence in general MASLD and cardiometabolic populations. They emphasise vegetables, fruit, whole grains, legumes, nuts, seeds, fish and unsaturated fats such as olive oil, while limiting sugar-sweetened beverages, ultra-processed foods, refined carbohydrates, saturated fat and processed meats [2,22,23]. In MASLD, this pattern is associated with improved cardiometabolic risk and reductions in liver fat, although superiority over all other energy-restricted healthy dietary patterns has not been established [16].

Dermatology-specific evidence is less direct. Observational studies in psoriasis have associated greater adherence to Mediterranean-style patterns with lower disease severity, but these studies do not establish that the dietary pattern improves hepatic outcomes in patients with concomitant psoriasis and MASLD [24,25]. Evidence in HS is predominantly observational and similarly does not establish liver-specific benefit [26]. Mediterranean principles should therefore be viewed as a practical, cardiometabolically favourable framework rather than a disease-specific treatment for the combined psoriasis/HS–MASLD population. Dietary and lifestyle modification also does not replace pharmacological or specialist management when indicated, particularly in patients with MASH, clinically significant fibrosis or major metabolic comorbidities.

### 3.2. Energy Restriction, Weight Loss and Physical Activity

Weight loss has a dose-dependent association with improvement in MASLD. Current consensus recommendations distinguish liver-related outcomes: approximately ≥5% body-weight reduction is associated with reduced liver fat, 7–10% with greater improvement in hepatic inflammation/MASH activity, and ≥10% is generally required for meaningful fibrosis improvement [2,16]. These thresholds should be regarded as treatment targets rather than guarantees of individual response.

Continuous daily caloric restriction—that is, a sustained daily energy deficit rather than restriction confined to designated fasting periods—and intermittent energy restriction both produce weight loss in general overweight or obese populations. Available evidence suggests broadly similar effects on body weight and selected metabolic outcomes, but formal equivalence and MASLD histology-specific comparative evidence are limited [16,27]. Low-carbohydrate and low-fat hypocaloric diets can both reduce liver fat; adherence, nutritional quality and sustained energy deficit are likely more important than a single macronutrient distribution for many patients [16,23].

In psoriasis, dietary weight reduction has shown modest adjunctive benefit among patients with overweight or obesity, including improvement in psoriasis severity and quality-of-life measures in some studies [24,25]. These dermatology studies generally did not evaluate MASLD-specific hepatic outcomes. Thus, weight reduction is reasonable when clinically indicated for metabolic health and may also benefit psoriasis, but the magnitude of hepatic benefit in patients with concomitant inflammatory skin disease has not been directly established. Regular physical activity should be encouraged alongside dietary intervention because exercise can improve cardiometabolic fitness and reduce hepatic steatosis even when weight loss is modest [16].

### 3.3. Dietary Quality, Macronutrients and Ultra-Processed Foods

No single ideal macronutrient distribution can be recommended for all patients with MASLD. Greater emphasis should instead be placed on overall dietary quality: replacing saturated fats with unsaturated fats, increasing dietary fibre, choosing minimally processed carbohydrate sources and limiting sugar-sweetened beverages and foods high in added fructose [16,23]. Higher fructose intake and dietary patterns rich in saturated fat and refined carbohydrate have been associated with adverse metabolic and liver outcomes, although much of this evidence is observational and should not be interpreted as proof of causality.

Ultra-processed foods (UPFs) are relevant to both metabolic and dermatological risk. MASLD guidance recommends minimising UPFs and sugar-sweetened beverages as part of a nutrient-dense eating pattern [2]. In a large prospective UK Biobank cohort, higher UPF consumption was associated with incident psoriasis, whereas replacing part of UPF intake with unprocessed or minimally processed foods was associated with lower psoriasis risk [28]. MASLD outcomes were not assessed in that psoriasis cohort. These findings support general dietary-quality advice but do not demonstrate that reducing UPFs directly improves liver disease in patients with psoriasis.

### 3.4. Lower-Certainty Elimination Diets in Hidradenitis Suppurativa

Dairy-free and brewer’s-yeast-free diets have been reported in HS, but the evidence is low certainty. Dairy avoidance is supported mainly by patient-reported survey data susceptible to recall and response bias, while brewer’s-yeast avoidance has been described in small uncontrolled cohorts in which concurrent interventions, including surgery, confound attribution of benefit [26,29]. These diets should not be routinely recommended. If an individual patient reports a reproducible dietary trigger, a time-limited elimination trial may be considered with dietetic supervision to minimise unnecessary restriction and nutritional inadequacy. No evidence establishes a liver-specific benefit of these elimination diets.

### 3.5. Cultural Adaptation and Practical Dietary Principles

**Figure 2:**
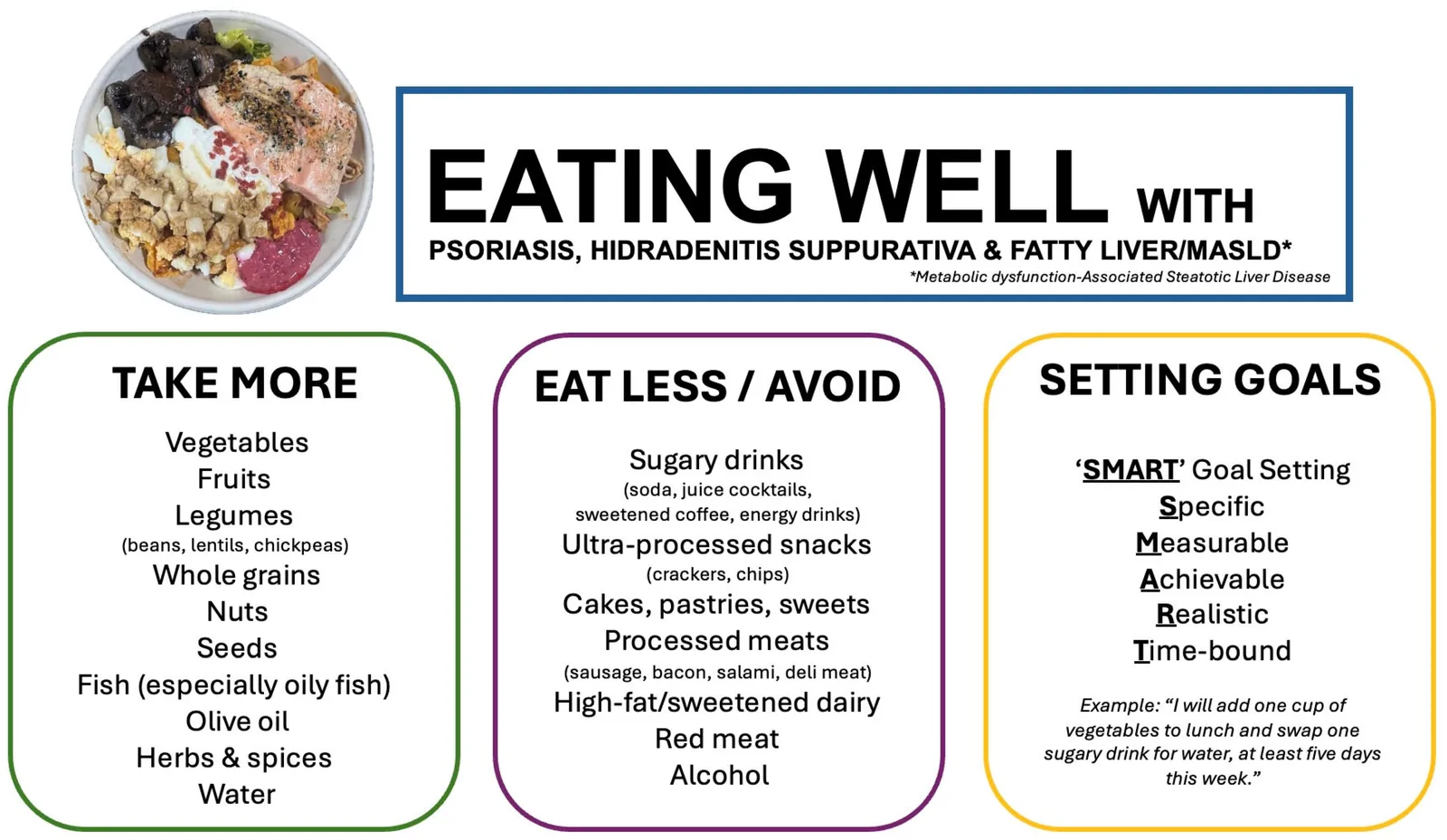
Patient-facing dietary guide for individuals with psoriasis, hidradenitis suppurativa, and metabolic dysfunction-associated steatotic liver disease (MASLD).

Dietary recommendations should be adapted to cultural preferences, food availability, cost, comorbidities and the patient’s capacity for sustained behavioural change. Mediterranean dietary principles can be implemented using locally available foods rather than reproducing a specific European menu; examples include locally available vegetables, legumes, whole grains, fish, nuts, seeds and plant oils rich in unsaturated fat. Family or household involvement may improve feasibility where meals are shared. Practical goals are often more useful than prescriptive meal plans—for example, replacing sugar-sweetened beverages with water, increasing vegetables or legumes, choosing minimally processed staples and reducing processed meats and snack foods. Figure 2 provides an example of a patient-facing dietary guide.

## 4. Nutritional Considerations During Incretin-Based Therapy

Incretin-based pharmacotherapy is increasingly relevant to patients with obesity, type 2 diabetes and MASLD, including patients encountered in dermatology practice. Because the focus of this review is nutritional management, pharmacological efficacy is summarised briefly and the discussion centres on dietary consequences and risk mitigation.

### 4.1. Clinical Context

Semaglutide, a glucagon-like peptide-1 receptor agonist (GLP-1RA), has demonstrated MASH resolution and improvement in fibrosis-related endpoints in phase 3 data and has received U.S. Food and Drug Administration accelerated approval for adults with noncirrhotic MASH and moderate-to-advanced fibrosis [1,30]. Other incretin-based agents include the dual glucose-dependent insulinotropic polypeptide (GIP)/GLP-1 receptor agonist tirzepatide, dual GLP-1/glucagon agonists and emerging triple agonists; these should not be collectively described simply as GLP-1RAs [31]. Direct evidence regarding their nutritional consequences specifically in patients with psoriasis or HS and MASLD remains sparse.

### 4.2. Reduced Intake, Protein Adequacy and Lean-Mass Preservation

Appetite suppression, early satiety and reduced energy intake contribute to the efficacy of incretin-based therapies but can also reduce dietary variety and protein intake [32,33]. Weight loss is accompanied by loss of both fat and lean mass, and concern is greatest in older or frail adults, patients with low baseline muscle mass, very rapid weight loss, low protein intake or little resistance exercise [34,35].

Higher protein targets, such as 1.2–1.6 g/kg/day, have been proposed during active weight reduction, but there is no consensus on whether actual, adjusted or ideal body weight should be used in individuals with obesity, and these targets have not been validated specifically in GLP-1-treated dermatology populations [33]. Protein goals should therefore be individualised according to body composition, renal function, age, total energy intake and clinical context. Protein-rich foods can be prioritised within the smaller volume of food tolerated, and resistance exercise should accompany adequate protein intake because protein supplementation alone is unlikely to preserve lean mass optimally [33,36].

### 4.3. Micronutrient Considerations

Direct prospective evidence linking incretin-based therapy to specific micronutrient deficiencies remains limited. Nutritional risk appears to arise principally from reduced food intake, reduced dietary diversity, gastrointestinal intolerance and pre-existing vulnerability rather than from an established drug-induced malabsorptive mechanism [32,33]. Potentially vulnerable patients include those with highly restrictive diets, persistent vomiting or diarrhoea, rapid or excessive weight loss, prior bariatric surgery, frailty or pre-existing nutritional deficiencies. Specific calorie thresholds and laboratory-monitoring schedules proposed in obesity guidance are expert-derived and should not be interpreted as validated GLP-1RA-specific thresholds for patients with psoriasis or HS.

Vitamin D deserves particular mention because low circulating 25-hydroxyvitamin D concentrations are common in psoriasis. However, association does not establish therapeutic benefit. A National Psoriasis Foundation systematic review found insufficient evidence to recommend oral vitamin D as a treatment for cutaneous psoriasis in patients with normal vitamin D status [24]. In a subsequent randomised trial, vitamin D supplementation increased serum 25-hydroxyvitamin D concentrations but did not significantly improve PASI or other clinical psoriasis outcomes compared with placebo [37]. Deficiencies should therefore be corrected for standard clinical indications rather than with the expectation of treating psoriasis itself.

Routine broad laboratory screening or universal supplementation during incretin therapy is not supported by prospective evidence. A risk-based approach is more appropriate: dietary assessment and targeted biochemical testing may be considered when symptoms, restricted intake, rapid weight loss, relevant comorbidities, prior deficiency or concomitant medications raise concern [33]. Nutrient-dense foods should remain the first-line strategy, with supplementation used to correct documented or strongly suspected inadequacy rather than replacing a balanced diet.

### 4.4. Gastrointestinal Adverse Effects

Nausea, vomiting, diarrhoea, constipation and early satiety are common during incretin-based therapy and may further compromise food and fluid intake [33,38]. Practical measures include smaller meals, slower eating, adequate hydration and temporary reduction in high-fat or very large meals when these exacerbate symptoms. Persistent symptoms, inability to maintain hydration or substantial unintended nutritional compromise should prompt clinical review and, where appropriate, dose adjustment or specialist input.

## 5. Dietary Supplements and Experimental Nutraceuticals

Evidence for supplements in MASLD is substantially weaker than evidence for dietary pattern and weight management, and no supplement has been adequately studied specifically in patients with concomitant psoriasis or HS and MASLD. Their inclusion in this review should not be interpreted as endorsement.

### 5.1. Vitamin E

Vitamin E has randomised histological data in selected patients with non-alcoholic steatohepatitis (the historical terminology used in the PIVENS trial). In PIVENS, natural-form RRR-α-tocopherol 800 IU/day improved histological disease activity in non-diabetic adults with biopsy-confirmed NASH, but did not demonstrate a consistent antifibrotic effect [39]. Observational data have suggested lower rates of hepatic decompensation and improved transplant-free survival among selected patients with advanced fibrosis, but residual confounding limits causal inference [40].

Guideline positions therefore remain cautious. The 2023 AASLD guidance stated that vitamin E may be considered in selected individuals without diabetes, whereas a more recent global MASLD/MASH consensus did not recommend routine use as a MASH-targeted therapy because phase 3 confirmation and consistent antifibrotic benefit are lacking [2,16]. Potential long-term harms, including the prostate-cancer signal reported in SELECT, should also be considered when prolonged high-dose therapy is contemplated [41].

### 5.2. Omega-3 Fatty Acids and Other Supplements

Omega-3 polyunsaturated fatty acids can improve hypertriglyceridaemia and may modestly affect selected surrogate markers of steatosis, but meta-analyses have not demonstrated consistent improvement in liver stiffness or histological outcomes [42,43]. They may therefore be used for an established indication such as hypertriglyceridaemia, but should not be presented as a treatment for MASH itself [16]. Silymarin has not demonstrated clinically meaningful histological benefit in MASH, and current guidance does not support its use as disease-specific therapy [16].

S-adenosylmethionine (SAMe) has antioxidant and methyl-donor functions, but the clinical literature is heterogeneous and includes few studies specific to metabolic liver disease. A systematic review reported possible improvements in liver biochemical parameters across mixed liver conditions, without evidence that SAMe produces MASH resolution, fibrosis regression or improved clinical outcomes [44]. Accordingly, SAMe should be regarded as an inadequately supported adjunct rather than an evidence-based MASLD treatment.

### 5.3. Experimental Compounds

Fatty acid esters of hydroxy fatty acids (FAHFAs) are endogenous bioactive lipids with anti-inflammatory and insulin-sensitising properties. Recent preclinical work with gut-retained synthetic FAHFA derivatives reduced gut-to-liver lipid flux and hepatic triglyceride accumulation in a diet-induced mouse model of MASLD [45]. Human efficacy, pharmacokinetics, safety and dosing have not been established. FAHFAs should therefore be described as experimental metabolic compounds rather than clinically validated nutraceuticals.

## 6. Potential Roles for Dermatologists Within Multidisciplinary Care

Dermatologists are well placed to recognise metabolic comorbidity because patients with psoriasis and HS may have repeated contact with dermatology services over many years. The appropriate scope of metabolic assessment nevertheless varies across healthcare systems, and liver risk stratification or pharmacological management should follow local pathways and clinician training.

A practical dermatology role is to recognise patients who may warrant further MASLD assessment, particularly those with psoriasis plus obesity, central adiposity, type 2 diabetes, dyslipidaemia, hypertension or psoriatic arthritis. Normal aminotransferase levels should not be used to exclude MASLD. Where local pathways support their use, non-invasive fibrosis tools such as FIB-4 and elastography can assist risk stratification, but interpretation and referral thresholds should follow relevant liver guidelines and institutional protocols [10,16].

Dermatologists can also provide general dietary and physical-activity advice consistent with MASLD care: emphasising minimally processed foods, vegetables, fruit, whole grains, legumes, fish and unsaturated fats; limiting sugar-sweetened beverages, processed meats and nutrient-poor ultra-processed foods; supporting appropriate weight reduction; and encouraging regular physical activity. Alcohol exposure should be assessed because metabolic dysfunction and alcohol can act synergistically in liver injury. Current steatotic-liver-disease nomenclature uses sex-specific alcohol-exposure thresholds to distinguish MASLD, MetALD and alcohol-related liver disease; these diagnostic classification thresholds should not be interpreted as recommended “safe” levels of alcohol intake [2,46].

For patients receiving incretin-based therapy, dermatologists should ask about appetite suppression, gastrointestinal symptoms, dietary restriction, rapid weight loss and functional decline rather than routinely ordering a fixed micronutrient panel for every patient. Targeted laboratory assessment and dietitian referral are appropriate when clinical risk factors are present. Referral to primary care, endocrinology, hepatology or a registered dietitian should be considered when metabolic disease is complex, fibrosis risk is elevated, nutritional adequacy is uncertain or weight-loss treatment requires more intensive management.

## 7. Communicating Uncertainty and Shared Decision-Making

Dietary advice should be framed around the strength and source of evidence. For patients with psoriasis, the association with MASLD is supported by epidemiological data, but most dietary recommendations remain extrapolated from general MASLD populations. For HS, both the epidemiological relationship with MASLD and the intervention evidence are less established. Clinicians should therefore avoid presenting a specific diet or supplement as proven to improve both skin and liver disease when direct evidence is absent.

Long-term adherence is also a central consideration. A dietary pattern that is nutritionally sound, culturally acceptable, affordable and sustainable is likely to be more useful than a theoretically optimal plan that cannot be maintained. Shared decision-making should incorporate comorbidities, medication effects, food preferences, socioeconomic constraints and the patient’s priorities. Where evidence is indirect or expert-derived, this uncertainty should be stated explicitly.

## 8. Current Gaps and Future Directions

Several evidence gaps remain. First, the prevalence and natural history of MASLD in HS require dedicated prospective study. Second, dietary-intervention trials in psoriasis and HS rarely include liver-specific outcomes; consequently, the hepatic effects of Mediterranean-style diets, weight loss, fasting strategies or other interventions in patients with concomitant inflammatory skin disease remain uncertain. Third, it is unknown whether effective control of cutaneous inflammation with biologic therapy modifies MASLD trajectory or interacts with lifestyle intervention. Fourth, the nutritional consequences of incretin-based therapy have not been prospectively characterised in dermatological populations. Finally, mechanistic work on microbiota and shared inflammatory pathways requires validation against clinically meaningful intervention outcomes.

Future studies should therefore prioritise well-characterised dermatology cohorts with standardised MASLD assessment, prespecified hepatic and dermatological outcomes, and clear reporting of weight change, diet quality, physical activity and concomitant systemic treatment. Such studies would allow clinicians to distinguish benefits attributable to weight loss and metabolic improvement from effects specific to a particular dietary pattern.

## 9. Conclusions

Psoriasis is consistently associated with a higher prevalence of MASLD, whereas the magnitude of MASLD risk in HS remains less well characterised. Shared metabolic and inflammatory abnormalities provide biological plausibility for overlap, but direct intervention evidence integrating skin disease, nutrition and liver outcomes is limited. Most dietary recommendations for these patients are therefore extrapolated from general MASLD evidence.

Mediterranean-style dietary principles, improved overall diet quality, appropriate energy restriction and sustained weight loss remain reasonable foundations of MASLD care. Weight-loss targets should be linked to the hepatic outcome of interest, with greater reductions generally required for improvement in MASH activity and fibrosis. Incretin-based therapy adds important nutritional considerations, particularly reduced intake, lean-mass preservation and risk-based assessment of nutritional adequacy. Dermatologists can contribute by recognising metabolic risk, providing general lifestyle counselling and facilitating multidisciplinary referral, while avoiding recommendations that exceed the available evidence or local scope of practice.

## Data Availability

No new data were created or analysed in this study. Data sharing is not applicable to this article.

## Supplementary Materials

The following supporting information can be downloaded at: https://www.mdpi.com/article/10.3390/nu18193253/s1, Table S1: Summary of dietary patterns, exposures and interventions relevant to MASLD, psoriasis and hidradenitis suppurativa, including source population and evidence category.

## Abbreviations

The following abbreviations are used in this manuscript:

BMI	Body mass index
cGAS	Cyclic GMP–AMP synthase
FAHFA	Fatty acid ester of hydroxy fatty acid
FIB-4	Fibrosis-4 index
GIP	Glucose-dependent insulinotropic polypeptide
GLP-1RA	Glucagon-like peptide-1 receptor agonist
HS	Hidradenitis suppurativa
IL	Interleukin
MASLD	Metabolic dysfunction-associated steatotic liver disease
MASH	Metabolic dysfunction-associated steatohepatitis
MetALD	Metabolic and alcohol-associated liver disease
PASI	Psoriasis Area and Severity Index
SAMe	S-adenosylmethionine
SCFA	Short-chain fatty acid
STING	Stimulator of interferon genes
TNF-α	Tumour necrosis factor-alpha
UPF	Ultra-processed food

## References

[1] Targher G., Valenti L., Byrne C.D. (2025). Metabolic Dysfunction–Associated Steatotic Liver Disease. N. Engl. J. Med..

[2] Younossi Z.M., Zelber-Sagi S., Lazarus J.V., Wong V.W.-S., Yilmaz Y., Duseja A., Eguchi Y., Castera L., Pessoa M.G., Oliveira C.P. (2025). Global Consensus Recommendations for Metabolic Dysfunction-Associated Steatotic Liver Disease and Steatohepatitis. Gastroenterology.

[3] Chan W.M.M., Yew Y.W., Theng T.S.C., Liew C.F., Oon H.H. (2020). Prevalence of Metabolic Syndrome in Patients with Psoriasis: A Cross-Sectional Study in Singapore. Singap. Med. J..

[4] Choi E.C.E., Phan P.H.C., Oon H.H. (2022). Hidradenitis Suppurativa: Racial and Socioeconomic Considerations in Management. Int. J. Dermatol..

[5] Untaaveesup S., Kantagowit P., Ungprasert P., Kitlertbanchong N., Vajiraviroj T., Sutithavinkul T., Techataweewan G., Eiumtrakul W., Threethrong R., Chaemsupaphan T. (2025). The Risk of Metabolic Dysfunction-Associated Steatotic Liver Disease in Moderate-to-Severe Psoriasis: A Systematic Review and Meta-Analysis. J. Clin. Med..

[6] Küçük K., Moreno C., Nijmi H., Daoud M., Mintoff D., Willaert F., Benhadou F. (2025). Identification of Risk Factors Associated with Metabolic Dysfunction-Associated Steatotic Liver Disease in Psoriatic Patients. Dermatology.

[7] James L., Diamond C., Al-Mossawi H., Andersson A., Pandya P., Shumbayawonda E., Firouzabadi L.I., Watson L., Savage L.J., Thomaides-Brears H. (2025). High Prevalence of MASLD in Psoriasis and Psoriatic Arthritis Assessed with Multiparametric Magnetic Resonance Imaging. Rheumatology.

[8] Chng W.Q., Liew J.J., Liew C.F., Muthiah M., Zheng M.H., Oon H.H. (2026). Hidradenitis Suppurativa and Psoriasis: Shared Immunological Links with Metabolic-Associated Steatotic Liver Disease. Front. Immunol..

[9] Lin Z., Shi Y.Y., Yu L.Y., Ma C.X., Pan S.Y., Dou Y., Zhou Q.J., Cao Y. (2024). Metabolic Dysfunction Associated Steatotic Liver Disease in Patients with Plaque Psoriasis: A Case–Control Study and Serological Comparison. Front. Med..

[10] Lai K.H.-T., Wong V.W.-S. (2026). Metabolic Dysfunction-Associated Steatotic Liver Disease in Asia: Implications on Management. Singap. Med. J..

[11] Eslam M., Chen F., George J. (2020). NAFLD in Lean Asians. Clin. Liver Dis..

[12] Fletcher J.M., Moran B., Petrasca A., Smith C.M. (2020). IL-17 in Inflammatory Skin Diseases Psoriasis and Hidradenitis Suppurativa. Clin. Exp. Immunol..

[13] Tan S., Liu M., Feng F., Li R., Tian R., Nie Z. (2024). Exploring the Pathogenesis and Immunological Profiles of Psoriasis Complicated with MASLD. PLoS ONE.

[14] Caro-Chang L.A., Fung M.A. (2024). Eosinophils in Psoriasis: A Systematic Review and Meta-Analysis Introducing a Study Quality Assessment Tool for Diagnostic Pathology Studies. J. Cutan. Pathol..

[15] Heitmann J., Frings V.G., Geier A., Goebeler M., Kerstan A. (2021). Non-Alcoholic Fatty Liver Disease and Psoriasis–Is There a Shared Proinflammatory Network?. J. Dtsch. Dermatol. Ges..

[16] Rinella M.E., Neuschwander-Tetri B.A., Siddiqui M.S., Abdelmalek M.F., Caldwell S., Barb D., Kleiner D.E., Loomba R. (2023). AASLD Practice Guidance on the Clinical Assessment and Management of Nonalcoholic Fatty Liver Disease. Hepatology.

[17] Aragonès G., González-García S., Aguilar C., Richart C., Auguet T. (2019). Gut Microbiota-Derived Mediators as Potential Markers in Nonalcoholic Fatty Liver Disease. BioMed Res. Int..

[18] Belinchón-Romero I., Bellot P., Romero-Pérez D., Herraiz-Romero I., Marco F., Frances R., Ramos-Rincón J.M. (2021). Non-Alcoholic Fatty Liver Disease Is Associated with Bacterial Translocation and a Higher Inflammation Response in Psoriatic Patients. Sci. Rep..

[19] Aguwa C., Enwereji N., Santiago S., Hine A., Kels G.G., McGee J., Lu J. (2023). Targeting Dysbiosis in Psoriasis, Atopic Dermatitis, and Hidradenitis Suppurativa: The Gut-Skin Axis and Microbiome-Directed Therapy. Clin. Dermatol..

[20] Wang B., Zhang Y., Lin L., Wang S., Yang S. (2026). Psoriasis: Microbiome Dysbiosis and Pathogenic Mechanisms. Front. Immunol..

[21] Wu J., Zhou Y., Zeng B., Wang Y., Wang C. (2026). Inside Out: How Cellular Localisation Shapes CGAS Functions in Health and Disease. Immunology.

[22] Tilg H., Petta S., Stefan N., Targher G. (2026). Metabolic Dysfunction–Associated Steatotic Liver Disease in Adults: A Review. JAMA.

[23] Cusi K., Abdelmalek M.F., Apovian C.M., Balapattabi K., Bannuru R.R., Barb D., Bardsley J.K., Beverly E.A., Corbin K.D., Elsayed N.A. (2025). Metabolic Dysfunction–Associated Steatotic Liver Disease (MASLD) in People With Diabetes: The Need for Screening and Early Intervention. A Consensus Report of the American Diabetes Association. Diabetes Care.

[24] Ford A.R., Siegel M., Bagel J., Cordoro K.M., Garg A., Gottlieb A., Green L.J., Gudjonsson J.E., Koo J., Lebwohl M. (2018). Dietary Recommendations for Adults with Psoriasis or Psoriatic Arthritis from the Medical Board of the National Psoriasis Foundation: A Systematic Review. JAMA Dermatol..

[25] Cintoni M., Palombaro M., Maramao F.S., Raoul P., Egidi G., Leonardi E., Bianchi L., Campione E., Rinninella E., Gasbarrini A. (2023). Metabolic Disorders and Psoriasis: Exploring the Role of Nutritional Interventions. Nutrients.

[26] Celik E., Bechara F.G., Stockfleth E., Ocker-Serger L., Abu Rached N. (2026). Dietary Patterns, Metabolic Pathways and Metainflammation in Hidradenitis Suppurativa: A Systematic Review. Front. Immunol..

[27] Grocic M., Nguyen M., Sanyal A.J. (2026). Current Trends in the Epidemiology of Obesity and the Association Between Obesity and Metabolic Liver Disease (MASLD/MASH). Diabetes Obes. Metab..

[28] Peng X., Li X., He J., He M., Ning N., Chen L., Yao P., Tang Y., Li Y. (2025). Ultra-Processed Food Consumption and the Risk of Psoriasis: A Large Prospective Cohort Study. Nutrients.

[29] Alikhan A., Sayed C., Alavi A., Alhusayen R., Brassard A., Burkhart C., Crowell K., Eisen D.B., Gottlieb A.B., Hamzavi I. (2019). North American Clinical Management Guidelines for Hidradenitis Suppurativa: A Publication from the United States and Canadian Hidradenitis Suppurativa Foundations: Part I: Diagnosis, Evaluation, and the Use of Complementary and Procedural Management. J. Am. Acad. Dermatol..

[30] U.S. Food and Drug Administration FDA Approves Treatment for Serious Liver Disease Known as ‘MASH’. https://www.fda.gov/drugs/news-events-human-drugs/fda-approves-treatment-serious-liver-disease-known-mash.

[31] Zhou X.D., Fan Q.Y., Byrne C.D., Targher G., Muthiah M.D., Huang D.Q., Chen Q.F., Noureddin M., Li W., Ratziu V. (2026). Combination Therapies for Metabolic Dysfunction-Associated Steatohepatitis: Challenges and Opportunities. Gut.

[32] Mogna-Peláez P., Guasch-Ferré M. (2026). Avoiding Malnutrition in the Era of GLP-1 Medications: Emerging Evidence and Opportunities for Integrated Nutrition Care. J. Nutr..

[33] Mozaffarian D., Aggarwal M., Alexander L., Apovian C.M., Bindlish S., Bonnet J., Butsch W.S., Christensen S., Gianos E., Gulati M. (2025). Nutritional Priorities to Support GLP-1 Therapy for Obesity: A Joint Advisory From the American College of Lifestyle Medicine, the American Society for Nutrition, the Obesity Medicine Association, and the Obesity Society. Am. J. Lifestyle Med..

[34] Bhandarkar A., Bhat S., Kapoor N. (2025). Effect of GLP-1 Receptor Agonists on Body Composition. Curr. Opin. Endocrinol. Diabetes Obes..

[35] Huang C.-F., Wu C.-H. (2025). Sarcopenic Obesity in the Asia-Pacific Region: Epidemiology, Risk Factors, and Management. Osteoporos. Sarcopenia.

[36] Mehrtash F., Dushay J., Manson J.A.E. (2025). Integrating Diet and Physical Activity When Prescribing GLP-1s-Lifestyle Factors Remain Crucial. JAMA Intern. Med..

[37] Jenssen M., Furberg A.S., Jorde R., Wilsgaard T., Danielsen K. (2023). Effect of Vitamin D Supplementation on Psoriasis Severity in Patients with Lower-Range Serum 25-Hydroxyvitamin D Levels: A Randomized Clinical Trial. JAMA Dermatol..

[38] Ho M.J., Liew C.F., Tan N.S., Morita A., Oon H.H. (2026). Glucagon-like Peptide-1 Receptor Agonists (GLP-1RAs) in Dermatology: Cutaneous Adverse Events and Emerging Efficacy in Inflammatory Skin Diseases. Expert Rev. Clin. Immunol..

[39] Sanyal A.J., Chalasani N., Kowdley K.V., McCullough A., Diehl A.M., Bass N.M., Neuschwander-Tetri B.A., Lavine J.E., Tonascia J., Unalp A. (2010). Pioglitazone, Vitamin E, or Placebo for Nonalcoholic Steatohepatitis. N. Engl. J. Med..

[40] Vilar-Gomez E., Vuppalanchi R., Gawrieh S., Ghabril M., Saxena R., Cummings O.W., Chalasani N. (2020). Vitamin E Improves Transplant-Free Survival and Hepatic Decompensation Among Patients with Nonalcoholic Steatohepatitis and Advanced Fibrosis. Hepatology.

[41] Klein E.A., Thompson I.M., Tangen C.M., Crowley J.J., Lucia M.S., Goodman P.J., Minasian L.M., Ford L.G., Parnes H.L., Gaziano J.M. (2011). Vitamin E and the Risk of Prostate Cancer: The Selenium and Vitamin E Cancer Prevention Trial (SELECT). JAMA.

[42] He X.X., Wu X.L., Chen R.P., Chen C., Liu X.G., Wu B.J., Huang Z.M. (2016). Effectiveness of Omega-3 Polyunsaturated Fatty Acids in Non-Alcoholic Fatty Liver Disease: A Meta-Analysis of Randomized Controlled Trials. PLoS ONE.

[43] Kim S.J., Cho S.H., Yun J.M. (2025). Omega-3 Polyunsaturated Fatty Acids and Nonalcoholic Fatty Liver Disease in Adults: A Meta-Analysis of Randomized Controlled Trials. Clin. Nutr..

[44] Baden K.E.R., McClain H., Craig E., Gibson N., Draime J.A., Chen A.M.H. (2024). S-Adenosylmethionine (SAMe) for Liver Health: A Systematic Review. Nutrients.

[45] Xie H., Cheng H.S., Ng J.X.J., Zhang S., Kim J.H.S., Tan S.H., Tan C.H., Tan N.S. (2026). A Gut-Liver Lipid Flux Checkpoint Mediates FAHFA Protection from MASLD. Pharmacol. Res..

[46] Sia J., Liew R.J., Liew C.F., Wong S.H., Tan W.R., Oon H.H., Patel V., Preedy V. (2026). Alcohol Flushing Syndrome (AFS), Alcohol-Associated Liver Disease (ALD) and Metabolic and Alcohol-Associated Liver Disease (MetALD): Features, Pathogenesis and Links with Cancer. Handbook of Alcohol Use and Alcohol Use Disorders: From Biology to Public Health.

