# Supplementary material for: Dietary and Nutritional Considerations for Metabolic Dysfunction-Associated Steatotic Liver Disease in Psoriasis and Hidradenitis Suppurativa: A Narrative Review
Source: Nutrients. 2026 Oct 2;18(19):3253. doi: 10.3390/nu18193253 (PMC13666952; doi:10.3390/nu18193253)
Supplement: Supplementary file 1 [file nutrients-18-03253-s001.zip › nutrients-4567595-supplementary.pdf]

Dietary pattern / exposure / intervention	Evidence type	Population / diagnostic context	Duration	Skin-disease-specific findings	MASLD / metabolic outcomes	Ref
Mediterranean dietary pattern	Guideline/consensus and narrative/systematic review synthesis	Adults with MASLD; patients with psoriasis or HS	Variable	Psoriasis: Greater adherence is associated with a lower likelihood and severity of psoriasis and may support symptom control. HS: Observational studies generally associate greater adherence with lower disease severity; evidence is predominantly cross-sectional or observational.	Preferred dietary pattern within first-line lifestyle management. Associated with improved insulin sensitivity and hepatic steatosis, with the strongest long-term cardiometabolic and mortality data among dietary patterns.	[22,23,25–27]
Caloric restriction and lifestyle-induced weight loss	Prospective biopsy study summarized in a clinical review; psoriasis randomized and interventional studies summarized in a narrative review	Adults with biopsy-confirmed MASH; adults with psoriasis and overweight/obesity	MASLD: 52 weeks; psoriasis: variable	In adults with psoriasis and overweight/obesity, adjunctive low-calorie diets and weight loss improved PASI and DLQI. Long-term adherence was a limitation, particularly with 800-1000 kcal/day regimens.	At least 5% weight loss reduces hepatic steatosis; 7-10% promotes MASH resolution; approximately 10% or more is generally needed for fibrosis improvement, although response varies.	[22,23,25]
Continuous versus intermittent caloric restriction	Narrative review citing systematic reviews/meta-analyses of randomized trials	Adults with overweight/obesity; not clearly MASLD-specific	Variable	No psoriasis- or HS-specific comparative outcomes were identified in the attached sources.	Intermittent and continuous caloric restriction appear similarly effective for weight loss and broadly similar insulin and body-fat outcomes. MASLD histology-specific comparative evidence is not established.	[27]
Low-carbohydrate versus low-fat hypocaloric diets	Clinical review and consensus synthesis of comparative dietary studies	Adults with MASLD	Variable	No psoriasis- or HS-specific comparative outcomes were identified in the attached sources.	Low-carbohydrate and low-fat hypocaloric diets have similar effectiveness in reducing liver fat and serum aminotransferase levels; several dietary approaches appear comparable for improving steatosis.	[22,23]
Ultra-processed food intake and incident psoriasis	Prospective cohort study (UK Biobank)	Adults aged 40-69 years without psoriasis at baseline	Median 12 years	The highest versus lowest UPF-consumption quartile was associated with increased incident psoriasis (HR 1.23, 95% CI 1.03-1.47). Replacing 20% of UPF weight with unprocessed or minimally processed food was associated with an 18% lower risk (HR 0.82, 95% CI 0.72-0.94).	MASLD outcomes were not assessed in this cohort.	[28]
Ultra-processed food-rich dietary patterns in MASLD	Consensus and narrative review synthesis	Adults with MASLD or at high metabolic risk	Variable	No skin-disease-specific outcomes were assessed in the cited MASLD sources.	Minimizing UPFs and sugar-sweetened beverages is recommended. UPF-rich patterns high in sugar and saturated fat are associated with greater risk of steatohepatitis and fibrosis progression, reported partly independently of total caloric intake.	[23,27]
Dairy avoidance in HS	Patient survey summarized in a clinical guideline and systematic review	Patients with HS	Not reported	Eighty-three percent reported some improvement and none reported worsening; however, response and recall bias were likely. Evidence is insufficient to recommend routine dairy avoidance.	No intervention-specific MASLD outcome data were identified.	[26,29]
Brewer's yeast avoidance in HS	Small uncontrolled cohort with concurrent surgery, summarized in a clinical guideline and systematic review	Patients with HS undergoing surgery plus yeast-exclusion diet	12 months	All participants improved and reported recurrence after consuming brewer's yeast, but concurrent surgery confounds attribution of benefit. Evidence is insufficient to recommend routine avoidance.	No intervention-specific MASLD outcome data were identified.	[26,29]

Abbreviations: CI, confidence interval; DLQI, Dermatology Life Quality Index; HR, hazard ratio; HS, hidradenitis suppurativa; MASH, metabolic dysfunction-associated steatohepatitis; MASLD, metabolic dysfunction-associated steatotic liver disease; N/A, not applicable; PASI, Psoriasis Area and Severity Index; RCT, randomized controlled trial; UPF, ultra-processed food.

Table S1. Summary of evidence on dietary patterns, dietary exposures, and nutritional interventions relevant to metabolic dysfunction-associated steatotic liver disease (MASLD), psoriasis, and hidradenitis suppurativa (HS).
